# Regional Brain Responses in Nulliparous Women to Emotional Infant Stimuli

**DOI:** 10.1371/journal.pone.0036270

**Published:** 2012-05-10

**Authors:** Jessica L. Montoya, Nicole Landi, Hedy Kober, Patrick D. Worhunsky, Helena J. V. Rutherford, W. Einar Mencl, Linda C. Mayes, Marc N. Potenza

**Affiliations:** 1 Department of Psychiatry, Yale University School of Medicine, New Haven, Connecticut, United States of America; 2 Yale Child Study Center, Yale University School of Medicine, New Haven, Connecticut, United States of America; 3 Haskins Laboratories, New Haven, Connecticut, United States of America; 4 Department of Neurobiology, Yale University School of Medicine, New Haven, Connecticut, United States of America; University of Missouri-Kansas City, United States of America

## Abstract

Infant cries and facial expressions influence social interactions and elicit caretaking behaviors from adults. Recent neuroimaging studies suggest that neural responses to infant stimuli involve brain regions that process rewards. However, these studies have yet to investigate individual differences in tendencies to engage or withdraw from motivationally relevant stimuli. To investigate this, we used event-related fMRI to scan 17 nulliparous women. Participants were presented with novel infant cries of two distress levels (low and high) and unknown infant faces of varying affect (happy, sad, and neutral) in a randomized, counter-balanced order. Brain activation was subsequently correlated with scores on the Behavioral Inhibition System/Behavioral Activation System scale. Infant cries activated bilateral superior and middle temporal gyri (STG and MTG) and precentral and postcentral gyri. Activation was greater in bilateral temporal cortices for low- relative to high-distress cries. Happy relative to neutral faces activated the ventral striatum, caudate, ventromedial prefrontal, and orbitofrontal cortices. Sad versus neutral faces activated the precuneus, cuneus, and posterior cingulate cortex, and behavioral activation drive correlated with occipital cortical activations in this contrast. Behavioral inhibition correlated with activation in the right STG for high- and low-distress cries relative to pink noise. Behavioral drive correlated inversely with putamen, caudate, and thalamic activations for the comparison of high-distress cries to pink noise. Reward-responsiveness correlated with activation in the left precentral gyrus during the perception of low-distress cries relative to pink noise. Our findings indicate that infant cry stimuli elicit activations in areas implicated in auditory processing and social cognition. Happy infant faces may be encoded as rewarding, whereas sad faces activate regions associated with empathic processing. Differences in motivational tendencies may modulate neural responses to infant cues.

## Introduction

During early development, pre-linguistic vocalizations, such as cries, and facial expressions are the primary means of infant communication. Both cries and facial expressions from the infant communicate salient information regarding their emotional states and needs and may elicit affection and nurturing from adults [1]. The interpretation and response to needs underlying infants’ sensory cues may significantly influence the infant’s development [2]; thus, the processing of the emotional content of infant stimuli is of developmental significance.

Utilizing auditory and visual sensory cues, functional magnetic resonance imaging (fMRI) studies have begun to examine mothers’ neural responses to emotional infant stimuli (e.g., [3], [4], [5], [6], [7], [8], [9], [10], [11], [12], [13], [14], [15]). Although auditory stimuli, like cries, may be experienced behaviorally differently than images of infants, considerable overlap is found in neural activation patterns [16], [17]. Specifically, regions such as the midbrain, hypothalamus, thalamus, basal ganglia, anterior cingulate cortex (ACC), and prefrontal cortex are commonly activated in fMRI studies of parental responses to infant cues, suggesting the involvement of motivation and reward circuitry [16], [17].

As individual differences in motivational tendencies may influence sensitivity to emotional stimuli and/or attachment processes, an exploratory examination of the relationship between brain activation patterns and individual differences in behavioral tendencies may be helpful in characterizing the neural responses to emotional infant stimuli. A prominent theory of behavioral tendencies predicts individual differences to engage or withdraw from emotionally or motivationally relevant stimuli [18], [19]. In this model, a behavioral activation system (BAS) exists to govern approach behavior toward rewarding stimuli, operating orthogonally to a behavioral inhibition system (BIS) that mediates withdrawal behavior from punishing stimuli. Measures such as the BIS/BAS scale [20] can be used to assess these tendencies. An improved understanding of how individual differences in behavioral inhibition and activation might relate to neural correlates of infant emotion processing could prove important in identifying features influencing adult-infant interactions.

In addition to BIS/BAS, evidence suggests that other factors may influence neural responding to infant stimuli. For example, comparisons of different stages in parenting (e.g., two to four weeks postpartum versus three to four months postpartum) have revealed differential patterns of brain activation, suggesting that experience with an infant over the initial months postpartum likely involves significant changes in responsivity to infant cues [13], [14]. As the experience of parenting may influence responses to infant stimuli, it is necessary to investigate the neural responses to infant stimuli in nulliparous women. Such investigations are important as they will not only inform studies of maternal responses to infant stimuli and help characterize shifts in maternal brain function, but also provide insight into a large group of women with more variable experiences with, and propensities towards, infants.

Therefore, in the current study, we sought to examine neural responses of nulliparous women to infant cries and faces of varying intensity and valence, respectively. Specifically, we investigated infant cries of differing distress (high, low) levels and infant faces of varying affect (happy, sad, neutral) in nulliparous women using fMRI. We hypothesized that both cry types, relative to a neutral auditory stimulus, would recruit regions previously implicated in response to cries, including the superior temporal gyrus (STG), insula, and cingulate cortices. Moreover, based on the perceived aversiveness of the cries, we predicted that high-distress cries, compared to low-distress cries, would be associated with relatively increased activity in these same regions. With regard to infant facial stimuli, we predicted that happy faces, compared to neutral ones, would activate regions associated with positive emotion and reward processing, including the ventral striatum and orbitofrontal cortex (OFC) [21], [22], [23], [24], [25], [26]. Sad faces, compared to neutral ones, were hypothesized to activate brain areas implicated in dysphoric and/or empathic responses such as the amygdala and cingulate cortex [27]. Using a BIS/BAS measure, we predicted that behavioral activation, which reflects responses to stimuli of reward and non-punishment [28], would correlate with activations related to rewarding stimuli such as happy infant faces. We further predicted that behavioral inhibition, which is associated with heightened arousal, passive avoidance, and anxiety [28], would correlate with regional brain activations related to more aversive stimuli, such as high- and low-distress cries.

## Methods

### Subjects

Nineteen native-English-speaking, right-handed nulliparous women gave informed written consent and participated in this study approved by the Yale Human Investigation Committee. All research was conducted in accordance with the Declaration of Helsinki. One subject had excessive motion in multiple fMRI runs and was excluded from analyses; another subject completed only four of seven functional runs and was also excluded. The remaining 17 subjects were between the ages of 19 and 29 (M = 22.7, SD = 2.9) years and were in good health with no history of psychiatric or neurological disorders, and had normal or corrected-to-normal vision. Racial and ethnic composition consisted of ten Caucasian, two Asian-American, two African-American, one Pacific-Islander, and two Hispanic women.

All subjects completed the BIS/BAS scale [20], a 24-item valid and reliable self-report questionnaire rated on a 4-point scale (strong agreement to strong disagreement) measuring behaviorally aversive (i.e., behavioral inhibition) and appetitive (i.e., behavioral activation) motivations. The BIS/BAS factors into four subscales, with one factor assessing inhibition (BIS) and three factors assessing activation. The three BAS subscales assess the pursuit of appetitive goals (BAS drive), tendency to seek rewarding experiences (BAS fun-seeking), and responsiveness to reward (BAS reward-responsiveness).

### Auditory Stimuli – Infant Cries

Cry stimuli were generated from stimuli described previously [29]. Cries were elicited from infants between the ages of 27 and 32 days who were without serious illness at birth and during their one-month checkup. Cries were recorded in the infants’ homes before the infants were fed and required no additional external stimulation. Detailed information about the recording procedure is reported elsewhere [29]. We used four two-second segments generated by two infants. The cries were categorized as either high- or low-distress, resulting in both a high- and low-distress exemplar from both infants. We used two exemplars for each level of distress to avoid measuring differences associated with the physical properties of one particular cry. Prior to imaging, the distress level of the cries was verified by an independent group of ten nulliparous female participants (ages 19 to 24 years) who rated the cries on a scale of 1 (calm) to 10 (distressed). High-distress cries were rated as significantly more distressed (M = 8.06, SD = 1.3) than low-distress cries (M = 3.54, SD = .82) (*t* = 11.52, *p*<.0001).

In addition to cries, subjects heard a “neutral” auditory stimulus, which consisted of a two-second segment of 1/f, or “pink” noise. Pink noise has a frequency of 1/f, indicating that the power spectral density is inversely proportional to the frequency. Pink noise was used as a neutral stimulus because it is not produced by a human and, as compared to white noise, is considered more naturalistic as it occurs in natural systems, speech, and music [30]. Additional information on the acoustic properties of the cries and neutral stimulus has been previously reported [4].

### Visual Stimuli

Photographs of infant faces between the ages of five and ten months were adapted from Strathearn and McClure [31] and were previously used by our group [4]. Twenty-one images from each of the six infants, resulting in a total of 126 images, were balanced for both gender and race (Caucasian and African American). The infant-face images displayed happy, neutral, and sad affective states. The size, luminance, and contrast for all face stimuli were standardized, and faces were presented on a black background. Prior to imaging, face stimuli were rated by an independent group of 11 participants on a scale of 1 (happy) to 10 (distressed) to assess the perceived affect level. A repeated measures ANOVA of the infant-face ratings on the three emotions (happy, neutral, sad) was significant (*F*(2, 20) = 146.43, *p*<.001). Pairwise comparisons showed that happy faces (M = 2.19, SD = 0.75) were rated as significantly less distressed (Mean difference = −1.55, SD = 1.15, *p* = .006) than neutral faces (M = 3.74, SD = 1.43). Neutral faces were rated as significantly less distressed (Mean difference = −4.16, SD = 1.28, *p*<.001) than sad faces (M = 7.90, SD = 0.34).

### Design

Stimuli were presented using E-Prime software (Version 1.2; Psychology Software Tools Inc., Pittsburgh, PA). The auditory stimuli were delivered via headphones with no visual display. The visual stimuli were displayed foveally at the fixation point for 1000 ms and followed by a fixation cross. Subjects received seven functional runs, each consisting of 42 trials (six trials of each condition of interest and six one-back memory trials). The conditions of interest were high-distress cry, low-distress cry, pink noise, happy face, sad face, and neutral face. Trials of all conditions were presented in a counter-balanced succession. The duration of the inter-trial-interval (ITI) was jittered (4000–14000 ms) to allow event-related analysis and to minimize stimulus expectation.

During each run, subjects were asked to attend to the stimulus sequence of faces and cries. A one-back memory task was included to maintain and assess subjects’ attention during the task and were modeled but not included in further analyses. On a small proportion of trials (14%), subjects were presented with a row of question marks and either a visual stimulus (infant face) was presented above the question marks or an auditory stimulus (cry or pink noise) was delivered via the headphones. The question marks cued the subject to make a yes/no decision via a stimulus response box as to whether the current stimulus was identical to the stimulus of the preceding trial (i.e., a one-back memory task). Analysis of catch trial data revealed a mean accuracy rate of 91.27±0.05% (mean ± SD).

### Data Acquisition

Data were acquired with a Siemens Trio 3T magnetic resonance imaging system (Siemens AG, Erlangen, Germany) using a standard 12-channel head coil. Localizer images were acquired for prescribing the functional image volumes, aligning the eighth slice parallel to the plane transecting the anterior and posterior commissures. Functional images were collected using a gradient echo, echoplanar sequence (repetition time [TR] = 2000 ms; echo time [TE] = 30 ms; flip angle [FA] = 80°, field of view [FOV] 20 cm×20 cm, 64×64 matrix, 3.4 mm 3.4 mm in-plane resolution, 4 mm slice thickness, 32 slices). Each stimulus run consisted of 163 volumes, including an initial rest period of 12 seconds (to achieve signal stability) that was removed from analyses. High-resolution structural images were also collected (sagittal MPRAGE acquisition, TR = 2530 ms; TE = 3.66 ms; FA = 7°; FOV = 25.6 cm×25.6 cm; number of excitations [NEX] = 256×256×1; 1 mm slice thickness, no gap; 176 slices).

### Image Analysis

Following prior published protocols [32], functional data were preprocessed using SPM5 (Wellcome Functional Imaging Laboratory, London, United Kingdom). Preprocessing included slice-time correction to the first slice of each volume; SPM5’s two-pass realign-to-mean strategy, which ultimately realigns all functional images to a mean functional image; coregistration of the anatomical image and the average of these realigned functional images; coregistration of all functional images using the parameters obtained from coregistration of the mean image; application of the SPM Unified Segmentation process to the anatomical scan, using prior information from the International Consortium for Brain Mapping (ICBM) Tissue Probabilistic Atlas and estimation of non-linear warping parameters [33]; warping the functional images to the Montreal Neurological Institute (MNI) template space; reslicing into isometric 3 mm×3 mm×3 mm voxels; and subsequent smoothing of functional images using a 6 mm Gaussian kernel. All functional runs were inspected for motion in excess of one voxel, for which one participant was excluded from the analysis.

Once the functional images were preprocessed, first-level robust regression was performed using the standard general linear model but with iteratively reweighted least squares using the bisquare weighting function for robustness [32], [34], as implemented in MATLAB 7.3 (Mathworks, Natick, MA; robust.m). Motion parameters and high-pass filter parameters were added as additional regressors of no interest. Once conditions were estimated using percent signal change for each participant, a second-level, random effects analysis was performed to estimate contrasts between conditions using NeuroElf (NeuroElf.net) and following our prior methods. To correct for multiple comparisons we then used a Monte Carlo simulation, which takes into account the voxel-wise and cluster-volume thresholds to establish family-wise error (FWE) correction. Only regions with corrected *p*<.05 (i.e., α<.05) threshold at an uncorrected voxel-level threshold of *p*<.01 at each tail and a cluster of 45 were considered to be significantly activated or deactivated in the whole-brain analysis. Whole-brain correlations were computed to assess the relationship between brain activation and behavioral inhibition and activation as assessed by the BIS/BAS. To adequately correct for the multiple comparisons conducted in the correlation analysis with multiple measures, we employed a conservative Bonferroni correction to both height and whole-brain level thresholds across 24 exploratory correlations. Clusters were considered significant at a FWE corrected *p*<.05 threshold and subsequently Bonferroni-corrected with a corrected *p*<.002 threshold (at an uncorrected voxel-level threshold of *p*<.0005 at each tail and a cluster of 17). Anatomical labels of all results were confirmed using the Talairach Daemon toolbox as well as manually, using a human brain atlas [35].

## Results

### Brain Activations to Infant Cries

**Figure 1:**
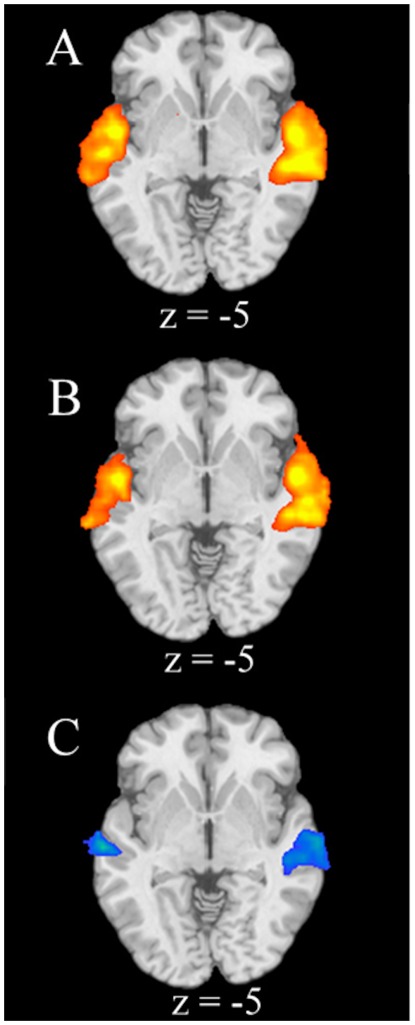
Regional Brain Activations during the Perception of Infant Cries. Axial slices of regional brain activations for a) low-distress cries versus pink noise, b) high-distress cries versus pink noise, c) and high-distress cries versus low-distress cries. Color on T1 template images from SPM5 indicates significant increases (red color) and decreases (blue color) in BOLD signal. The right side of the brain is on the right. The number under each brain image indicates z-axis coordinates of the image in the MNI (Montreal Neurological Institute) template space. The only voxels displayed on the brain images are regions with corrected p<.05 threshold at an uncorrected voxel-level threshold of p<.01 at each tail and a cluster of 45.

When comparing low-distress cries to pink noise, increased activation was observed in bilateral STG, right middle temporal gyrus (MTG), bilateral precentral and postcentral gyri, right inferior parietal lobe (IPL), left superior and medial frontal gyri (SFG and MFG), left putamen and left claustrum. Relatively diminished activation was observed in left caudate and right MFG/OFC. When comparing high-distress cries to pink noise, increased activation was observed in bilateral STG, right MTG, right precentral and postcentral gyri, right SFG, right MFG, right inferior frontal gyrus (IFG), bilateral amygdala, and left culmen. Relatively diminished activation was observed in the right STG and right insula. When comparing high-distress cries to low-distress cries, diminished activation was observed in bilateral STG, right MTG, left IPL, right superior occipital gyrus, and left precuneus; no regions showed increased activation (Table 1; Figure 1).

**Table 1:** Regional Brain Activations during the Perception of Infant Cries.

Contrast	Region	k	max	x	y	z
Low Distress Cry > Pink Noise						
	L. Superior Temporal Gyrus (BA 42)	979	11.75	−64	−29	7
	R. Middle Temporal Gyrus	1507	10.26	58	−32	2
	L. Postcentral Gyrus (BA 3)	248	5.21	−53	−12	49
	L. Medial frontal gyrus (BA 6)	203	4.56	−7	−3	50
	L. Lentiform Nucleus (Putamen)	72	4.38	−22	1	10
Pink Noise > Low Distress Cry						
	L. Caudate (Caudate Body)	84	4.76	−25	−15	27
	R. Middle Frontal Gyrus/Orbitofrontal gyrus (BA 11)	77	4.40	38	45	−17
High Distress Cry > Pink Noise						
	R. Superior Temporal Gyrus (BA 22)	1083	10.11	57	−8	1
	L. Superior Temporal Gyrus (BA 22)	665	9.01	−54	−9	3
	L. Culmen	45	4.35	−17	−51	−17
	R. Precentral Gyrus (BA 6)	155	4.15	46	−9	37
	R. Superior Frontal Gyrus (BA 6)	120	3.98	7	11	49
	L. Superior Temporal Gyrus (BA 38)	45	3.95	−34	5	−16
	R. Medial Frontal Gyrus (BA 6)	60	3.65	7	−18	53
Pink Noise > High Distress Cry						
	R. Superior Temporal Gyrus (BA 39)	128	5.22	51	−55	24
Low Distress Cry > High Distress Cry						
	L. Superior Temporal Gyrus	239	5.91	−64	−20	1
	R. Superior Temporal Gyrus (BA 22)	406	5.44	67	−33	13
	R. Middle Temporal Gyrus (BA 39)	93	5.32	50	−76	28
	L. Precuneus (BA 7)	46	3.49	−9	−52	38

All clusters in this table were generated at FWE corrected p<.05 threshold at an uncorrected voxel-level threshold of p<.01 at each tail and a cluster of 45. Abbreviations: BA: Brodmann area; L: left; R: right, k: number of voxels in the cluster.

### Brain Activations to Infant Faces

**Figure 2:**
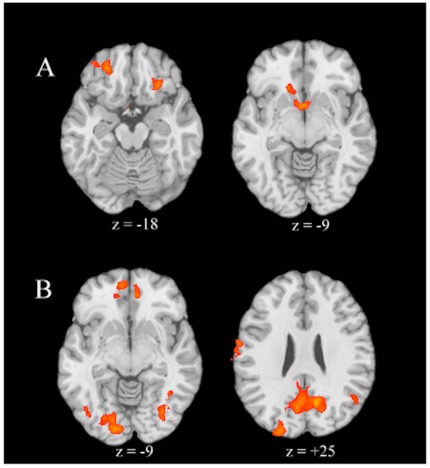
Regional Brain Activations during the Perception of Infant Faces. Axial slices of regional brain activations for a) happy versus neutral infant faces and b) sad versus neutral infant faces. Color on T1 template images from SPM5 indicates significant increases (red color) and decreases (blue color) in BOLD signal. The right side of the brain is on the right. The number under each brain image indicates z-axis coordinates of the image in the MNI (Montreal Neurological Institute) template space. The only voxels displayed on the brain images are regions with corrected p<.05 threshold at an uncorrected voxel-level threshold of p<.01 at each tail and a cluster of 45.

For happy versus neutral infant faces, greater activation was observed in left ventral striatum, left caudate head, left ventromedial prefrontal cortex (vmPFC)/OFC, and right IFG. Relatively diminished activation was observed in left cingulate gyrus, bilateral precentral gyrus, right SFG, right STG, left supramarginal gyrus, and left insula. For sad versus neutral infant faces, greater activation was observed in bilateral precuneus, left cingulate gyrus, right MTG, bilateral middle and inferior occipital gyri, right fusiform gyrus (FG), left precentral gyrus, left IPL, left lingual gyrus, right SFG, bilateral MFG, right IFG/OFC, and left ACC. Relatively reduced activation was observed in the left insula, left transverse temporal gyrus, and right STG. For happy versus sad faces, relatively greater activation during the presentation of sad faces was observed in the right IFG, bilateral FG, right STG, right supramarginal gyrus, right cuneus, left MTG, left middle occipital gyrus, right precentral gyrus, and right MFG; no regions demonstrated greater activation for the presentation of happy faces relative to sad faces (Table 2; Figure 2).

**Table 2:** Regional Brain Activations during the Perception of Infant Faces.

Contrast	Region	k	max	x	y	z
Happy Face > Neutral Face						
	L. Ventral striatum (BA 25)	72	4.80	−3	3	−6
	L. Ventromedial Prefrontal Cortex/Orbitofrontal Gyrus (BA 11)	45	4.69	−25	43	−21
	R. Inferior Frontal Gyrus (BA 47)	45	3.90	30	23	−13
Neutral Face > Happy Face						
	L. Cingulate Gyrus (BA 24)	77	4.47	−3	−14	41
	R. Superior Temporal Gyrus (BA 22)	98	4.40	53	−47	15
	L. Supramarginal Gyrus (BA 40)	49	4.31	−52	−43	33
	L. Insula (BA 13)	58	4.28	−40	−41	18
	L. Precentral Gyrus (BA 4)	50	3.78	−31	−18	56
Sad Face > Neutral Face						
	R. Precuneus (BA 31)	396	5.95	13	−67	20
	R. Middle Temporal Gyrus (BA 39)	84	4.91	53	−63	19
	R. Middle Occipital Gyrus (BA 18)	157	4.62	33	−84	5
	L. Precentral Gyrus (BA 6)	50	4.54	−65	−5	35
	L. Middle Occipital Gyrus (BA 19)	313	4.44	−28	−85	13
	R. Superior Frontal Gyrus (BA 10)	51	4.14	25	51	0
	R. Inferior Frontal Gyrus/Orbitofronal gyrus (BA 11)	79	3.99	11	42	−15
Neutral Face > Sad Face						
	L. Insula (BA 13)	62	4.95	−53	−38	14
	R. Superior Temporal Gyrus	48	3.71	62	−21	5
Sad Face > Happy Face						
	R. Inferior Frontal Gyrus (BA 45)	64	5.88	55	27	13
	R. Fusiform gyrus (BA 19)	47	5.06	37	−62	−4
	R. Superior Temporal Gyrus (BA 39)	89	4.82	43	−52	15
	R. Cuneus (BA 18)	83	4.69	19	−72	15
	L. Middle Temporal Gyrus (BA 37)	87	4.35	−40	−63	−1
	L. Middle Occipital Gyrus (BA 19)	64	4.11	−31	−82	6
	R. Precentral Gyrus (BA 6)	50	3.88	10	−24	64

All clusters in this table were generated at FWE corrected p<.05 threshold at an uncorrected voxel-level threshold of p<.01 at each tail and a cluster of 45. Abbreviations: BA: Brodmann area; L: left; R: right, k: number of voxels in the cluster.

### BIS/BAS Scores and Correlations with Brain Activity

The mean ± SD scores of the 17 subjects on the BIS/BAS scale components were 22.12±2.76 for the BIS, 10.76±2.22 for BAS drive, 11.29±1.93 for BAS fun seeking, and 17.35±1.54 for BAS reward-responsiveness. These scores fall within the standard mean score range for healthy subjects [36].

**Figure 3:**
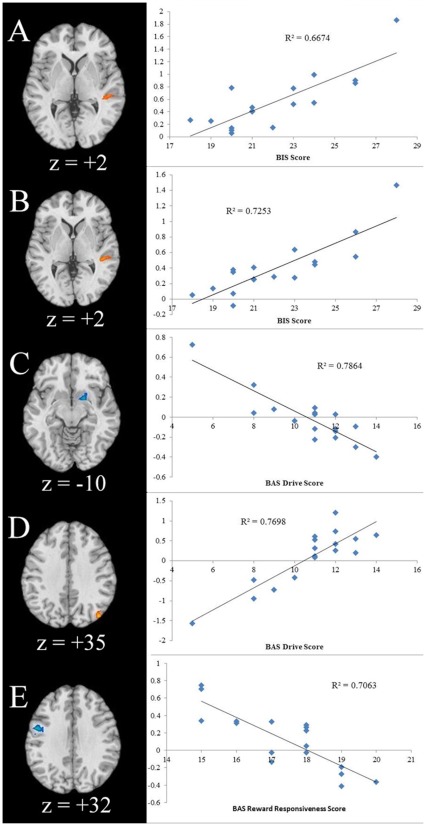
Regional Brain Activations during the Perception of Infant Cries and Faces Correlated with Behavioral Measures of Motivation as Assessed by BIS/BAS Subscales. a) Axial slice of regional brain response for low-distress cry versus pink noise that correlates with scores on the BIS scale. b) Axial slice of regional brain response for high-distress cry versus pink noise that correlates with scores on the BIS scale. c) Axial slice of regional brain response for high-distress cry versus pink noise that correlates with scores on the BAS drive scale d) Axial slice of regional brain response for sad versus neutral infant faces that correlates with scores on the BAS drive scale. e) Axial slice of regional brain response for low-distress cry versus pink noise that correlates with scores on the BAS reward-responsiveness scale. Color on T1 template images from SPM5 indicates significant increases (red color) and decreases (blue color) in BOLD signal. The right side of the brain is on the right. The number under each brain image indicates z-axis coordinates of the image in the MNI (Montreal Neurological Institute) template space. The only voxels displayed on the brain images are regions with corrected p<.002 threshold at an uncorrected voxel-level threshold of p<.0005 at each tail and a cluster of 17.

The scores on the BIS and two BAS subscales (drive and reward-responsiveness) were correlated with brain activation contrasts. BIS scores positively correlated with right STG activity in both the comparisons of high-distress cries versus pink noise and low-distress cry versus pink noise. The BAS drive subscale scores inversely correlated with activations in the: 1) right putamen, right caudate extending into the thalamus, right lateral globus pallidus, and left medial globus pallidus in the contrast between high-distress cries and pink noise; and 2) left angular gyrus in the contrast between high-distress cries and low-distress cries. The BAS drive subscale scores positively correlated with right superior occipital gyrus in the contrast between sad and neutral faces. BAS reward-responsiveness scores inversely correlated with left precentral gyral activation in the contrast between low-distress cries and pink noise (Table 3; Figure 3).

**Table 3:** Regional Brain Activations during the Perception of Infant Cries and Faces Correlated with Behavioral Measures of Motivation as Assessed by BIS/BAS Subscales.

Contrast	Region	k	max	x	y	z
High Distress Cry > Pink Noise Correlated with BIS						
	R. Superior Temporal Gyrus (BA 41)	25	0.83	45	-37	4
Low Distress Cry > Pink Noise Correlated with BIS						
	R. Superior Temporal Gyrus (BA 41)	17	0.81	48	-34	7
High Distress Cry > Pink Noise Correlated with BAS Drive						
	R. Lentiform Nucleus (Putamen)	19	-0.86	19	0	8
	R. Caudate (Caudate Body)	33	-0.85	10	14	16
	R. Lentiform Nucleus (Lateral Globus Pallidus)	18	-0.83	19	0	-4
	L. Lentiform Nucleus (Medial Globus Pallidus)	17	-0.83	-9	1	3
High Distress Cry > Low Distress Cry Correlated with BAS Drive						
	L. Angular Gyrus (BA 39)	19	-0.83	-55	-65	37
Sad Face > Neutral Face Correlated with BAS Drive						
	R. Superior Occipital Gyrus (BA 19)	20	0.87	45	-77	28
Low Distress Cry > Pink Noise Correlated with BAS Reward						
	L. Precentral Gyrus (BA 6)	37	-0.84	-51	-5	30

All clusters in this table were generated at FWE corrected *p*<.002 threshold at an uncorrected voxel-level threshold of *p*<.0005 at each tail and a cluster of 17. Abbreviations: BA: Brodmann area; L: left; R: right, k: number of voxels in the cluster.

## Discussion

The current study used fMRI to examine the neural correlates of how nulliparous women respond to emotional infant stimuli, specifically cries of varying distress levels and facial expressions of varying affect. Overall, regions activated in response to cries in nulliparous women (e.g., the STG and MTG) are consistent with those identified in cry processing in previous studies of both non-parents and parents [6], [11], [37]. For the face stimuli, we observed different regional brain activations in response to sad and happy infant faces. Regions such as the vmPFC, OFC, and ACC, which are commonly activated in fMRI studies of parental responses to infant cues, demonstrated activation during the presentation of infant faces [16], [17]. Furthermore, neural responses to the cry and face contrasts correlated with self-reported measures of behavioral inhibition and activation suggesting that neural responses to infant stimuli vary as a function of motivational approach and avoidance tendencies.

### Regional Brain Activations during the Perception of Low- and High-Distress Cries Relative to the Control Stimulus

We found increased activation to low-distress cries relative to the control stimulus in bilateral STG, right MTG, right IPL, and bilateral precentral and postcentral gyri. Similarly, high-distress cries relative to pink noise identified increased activation in bilateral STG, right MTG, and bilateral precentral and postcentral gyri. Findings in STG and frontal cortices are common in fMRI paradigms utilizing infant cry stimuli and may reflect auditory processing and social cognition [38], [39]. Several fMRI studies have linked activations of STG and IPL to representations of others’ intentions and mental states [40], [41]. Thus, activation in these areas during the perception of cries may reflect an attempt to understand the emotional states associated with cries of varying distress levels. The STG has also demonstrated increased activity in response to angry speech relative to neutral speech [42], [43]. Therefore, activation in STG may reflect the aversive nature of the cries.

### Regional Brain Activations during the Perception of Low- Relative to High-Distress Cries

Nulliparous women demonstrated greater activation for low-distress relative to high-distress cries in bilateral STG, right MTG and left IPL. Increased activation in auditory-processing regions for low- relative to high-distress cries may reflect the greater acoustic variability in the low-distress cries. Specifically, low-distress cries tend to have more numerous shorter bouts whereas high-distress cries tend to have fewer bouts and fewer breaths (see Appendix for cry characteristics). Accordingly, low-distress cries might be considered more complex and may generate relatively increased STG and MTG activation. From a behavioral perspective, high-distress cries may produce more unequivocal responses in adults (e.g., “The infant is clearly distressed and needs immediate attention.”), whereas low-distress cries may produce more complex, and potentially ambiguous, behavioral responses as the adult attempts to understand the cries’ meanings (e.g., “How greatly distressed is the infant? Will the cries cease without my attention?”). The potentially equivocal nature of these responses may relate to the observed increased insular activation, which has been associated with decision-making processes and empathy [44], [45]. Additionally, the greater recruitment of brain regions during low-distress cries relative to high-distress cries may stem from differential previous experiences of the nulliparous women with infants, which was not assessed in this study. Further research on the relationship between specific acoustic properties of cries and the neural and emotional responses they generate is necessary for understanding the differential responses to cries of varying properties.

### Regional Brain Activations during Viewing of Happy Faces

Consistent with our hypothesis and findings from previous studies involving the processing of infant visual stimuli [3], [7], [8], [12], viewing of happy infant faces compared to neutral ones engaged the OFC. The OFC contributes importantly to maternal “reward” circuitry [3], [12], and increased activation in this region may reflect the rewarding nature of a happy infant face, which may help elicit care-giving behaviors. Considered a component of the brain’s “reward system,” the OFC receives ascending dopamine projections from the ventral tegmental area (VTA) [23], [46]. Studies with pleasant visual, tactile, and olfactory stimuli have found increased activation in the OFC that depends on the pleasantness rather than the intensity of stimulation [47], [48]. The OFC is therefore considered to have a critical role in representing the reward value of a stimulus. Greater activation for happy faces was also seen in the striatum, a structure receiving projections from the VTA and OFC [49] and implicated in reward-related learning and motivated behaviors [22], [50], [51]. The increased striatal activation in nulliparous women may relate to the coding of happy infant affect as a positive sensory cue.

### Regional Brain Activations during Viewing of Sad Faces

For the sad versus neutral face contrast, activation was observed in the precuneus, cuneus, and left posterior cingulate cortex (PCC). Both the precuneus and PCC have been implicated in the processing of sad adult faces [27] and show greater activation when adults evaluate their own or other’s emotional states [52]. A longitudinal neuroimaging study of depressed patients found differential brain activations according to depression status [27], suggesting that areas involved in the discernment of negative affective facial expressions may relate to dysphoric response patterns. The PCC has also been implicated in stress responses [53], suggesting that stress neurocircuitry may be activated by sad faces. Alternatively, the activation of the precuneus and PCC may indicate that nulliparous women engage in the attribution of emotion while viewing sad infant visual stimuli, as the PCC has been associated with evaluating the affective valence of external stimuli [54], and the precuneus has been implicated in empathic processes [55].

Sad faces also activated the ACC, a region involved in the processing of emotional information [56]. Data implicate the ACC in attending to, and regulating, arousal associated with affective states [56], as increased blood flow has been reported in dorsal and rostral regions of the ACC when attending to subjective emotional states and experiences [57], [58]. Regions along the border between the rostral ACC and the mPFC have been associated with theory of mind tasks, such as the ability to infer mental states of others [59]. The increased activation in ACC therefore suggests that the nulliparous women in this study may have engaged in social and emotive processing while viewing the sad infant facial stimuli.

### Regional Brain Activations during Viewing of Sad-Relative-to-Happy Faces

For the comparison of sad versus happy faces, the right IFG, bilateral FG, and right cuneus demonstrated increased activity. Both the IFG and FG have been widely implicated within circuitry involved in the processing of adult emotional faces and are considered as “core” regions of emotional face processing [60], [61]. The FG has been implicated in the processing of facial stimuli [62], including in learning affective values of faces [63], with greater FG activation observed to faces of negative affect [64]. The precuneus has also been implicated in the processing of adult emotional faces, particularly in response to sad faces [65]. Precuneus response to emotional faces appears influenced by individual genetic variation [66] suggesting the value of face perception investigations of individual differences. Together, the findings suggest that the neural underpinnings of infant emotional face processing share similarities with those underlying adult emotional face processing and that that individual differences are important to consider in the processing of facial stimuli.

### Regional Brain Activations and Individual Differences in Behavioral Inhibition and Activation

Our findings suggest that individual differences in motivational tendencies may influence neural correlates underlying the processing of infant emotional cues. Specifically, higher self-reported behavioral inhibition was related to greater activation in right STG during the perception of low-distress cries relative to pink noise, as well as high-distress cries relative to pink noise. The BIS measure assesses responsiveness to signals of negative outcomes, particularly tendencies to inhibit behavior that may result in undesirable consequences (e.g., “If I think something unpleasant is going to happen, I usually get pretty worked up.”). The recruitment of right STG during the perception of low- and high-distress cries preferentially in women with high BIS scores may therefore relate to the aversive nature of cries, with individuals more prone to behavioral inhibition demonstrating a greater STG response.

Higher self-reported behavioral drive was associated with greater activation in the right superior occipital gyrus when viewing sad versus neutral faces. The occipital cortex, including the superior occipital gyrus, has been linked to affective processing, with occipital cortical activity correlating with poor social adjustment and impaired social cognition in individuals with psychotic disorders [67]. Thus, the current findings relating behavioral drive to superior occipital gyral activation during viewing of sad faces not only implicates a region implicated in social processing in a population often characterized by poor motivation drive and interpersonal difficulties, but also suggests that early visual processing may be particularly relevant to responses to sad infant facial cues in behaviorally driven individuals.

In the current study, individuals with higher reward-responsiveness showed lower activity in the left precentral gyrus when listening to low-distress cries compared to pink noise. The precentral gyrus, involved in motoric responding, has been implicated in the processing of rewarding and aversive stimuli. For example, healthy subjects as compared to individuals with borderline personality disorder, a condition characterized by emotional dysregulation, showed greater recruitment of the precentral gyrus during responses to aversive as compared to neutral stimuli [68]. Healthy women but not those with bulimia nervosa showed increased activation of the precentral gyrus in anticipation and receipt of a milkshake reward [69]. Thus, these findings suggest that individual differences in precentral gyral activations to aversive and rewarding cues may have important clinical implications. The current findings suggest that individual differences in both approach and avoidance motivational tendencies are related to neural activations involved in attentional and emotional processing. The extent to which these behavioral and neural measures relate to specific aspects of adult-infant interactions requires additional investigation.

### Limitations, Strengths, and Future Directions

Several limitations exist. First, the facial stimuli were derived solely from infants. Future investigations involving facial stimuli from individuals of varying ages may be helpful in elucidating how brain responses may be modulated by the physical maturity of facial features being viewed. Furthermore, the cries were gathered solely from newborn infants, limiting the possibility of having a comparable happy auditory condition such as laughter. Additionally, the age difference of the infants used for the cry and face stimuli makes comparisons between the two sensory domains difficult. However, we did find increased activation in precuneus, right MTG, left precentral gyrus, and left IPL for sad faces relative to neutral ones, as well as for cries relative to pink noise. Future fMRI investigations are needed to continue identifying regions activated across these sensory modalities. Second, the subjects in the study were healthy nulliparous women of childbearing age. Information regarding subjects’ desire and plans to be in a caretaker role, as well as the degree of their present interaction with infants, may help to further account for individual differences in the processing of infant stimuli. Additionally, studies of childbearing women could examine how neural responses to infant affective cues may change in healthy mothers at varying times postpartum. Third, the study excluded men. Examination of similarly aged men and parents of both sexes could investigate potential influences of sex and parenthood, respectively. Fourth, the study involved healthy subjects. Future studies of mothers and nulliparous women in whom parent-child interactions may become impaired, such as during maternal depression and substance abuse, could help investigate processes of particular relevance to the health of vulnerable youth. Despite these limitations, the findings provide initial insight into the neural processing of infant cues in nulliparous women and how individual differences in motivational tendencies relate to brain responses to infant stimuli.

In summary, the current study provides an initial examination of how emotional infant stimuli are perceived by healthy, nulliparous women. Cries of varying distress levels differentially recruited regions associated with auditory and empathic processing. With regard to the visual infant stimuli, our findings suggest that happy faces are encoded as rewarding stimuli in the brain, whereas sad faces induce increased activation in regions associated with empathic processing. The study is also the first to investigate appetitive and aversive motivational tendencies in relationship to the processing of infant emotional cues, and the findings suggest a relationship between individual differences in motivational tendencies and brain response patterns to infant cues. These findings also indicate the utility of this approach to investigate a broader range of individual differences with respect to neural activations and their clinical correlates in response to infant stimuli.
